# Proteomic analysis reveals response of differential wheat (*Triticum aestivum* L.) genotypes to oxygen deficiency stress

**DOI:** 10.1186/s12864-018-5405-3

**Published:** 2019-01-18

**Authors:** Rui Pan, Dongli He, Le Xu, Meixue Zhou, Chengdao Li, Chu Wu, Yanhao Xu, Wenying Zhang

**Affiliations:** 1 Hubei Collaborative Innovation Center for Grain Industry/ School of Agriculture, Yangtze University, Jingzhou, 434025 China; 2 College of Life Sciences, Hubei University, Wuhan, 430074 China; 3 Tasmanian Institute of Agriculture, University of Tasmania, Private Bag 1375, Prospect, Hobart, Tasmania 7250 Australia; 4 Western Barley Genetics Alliance, School of Veterinary and Life Sciences (VLS), Murdoch University, Murdoch, WA Australia; 5 College of Horticulture and Gardening, Yangtze University, Jingzhou, 434025 China

**Keywords:** *Triticum aestivum* L., Hypoxic stress, Waterlogging tolerance, Proteomics

## Abstract

**Background:**

Waterlogging is one of the main abiotic stresses that limit wheat production. Quantitative proteomics analysis has been applied in the study of crop abiotic stress as an effective way in recent years (e.g. salt stress, drought stress, heat stress and waterlogging stress). However, only a few proteins related to primary metabolism and signal transduction, such as UDP - glucose dehydrogenase, UGP, beta glucosidases, were reported to response to waterlogging stress in wheat. The differentially expressed proteins between genotypes of wheat in response to waterlogging are less-defined. In this study, two wheat genotypes, one is sensitive to waterlogging stress (Seri M82, named as S) and the other is tolerant to waterlogging (CIGM90.863, named as T), were compared in seedling roots under hypoxia conditions to evaluate the different responses at proteomic level.

**Results:**

A total of 4560 proteins were identified and the number of differentially expressed proteins (DEPs) were 361, 640, 788 in S and 33, 207, 279 in T in 1, 2, 3 days, respectively. These DEPs included 270 common proteins, 681 S-specific and 50 T-specific proteins, most of which were misc., protein processing, DNA and RNA processing, amino acid metabolism and stress related proteins induced by hypoxia. Some specific proteins related to waterlogging stress, including acid phosphatase, oxidant protective enzyme, S-adenosylmethionine synthetase 1, were significantly different between S and T. A total of 20 representative genes encoding DEPs, including 7 shared DEPs and 13 cultivar-specific DEPs, were selected for further RT-qPCR analysis. Fourteen genes showed consistent dynamic expression patterns at mRNA and protein levels.

**Conclusions:**

Proteins involved in primary metabolisms and protein processing were inclined to be affected under hypoxia stress. The negative effects were more severe in the sensitive genotype. The expression patterns of some specific proteins, such as alcohol dehydrogenases and S-adenosylmethionine synthetase 1, could be applied as indexes for improving the waterlogging tolerance in wheat. Some specific proteins identified in this study will facilitate the subsequent protein function validation and biomarker development.

**Electronic supplementary material:**

The online version of this article (10.1186/s12864-018-5405-3) contains supplementary material, which is available to authorized users.

## Background

High rainfall, combined with poor soil structure, usually causes severe waterlogging which is one of the main global abiotic stresses limiting crop production. About ten million ha of the wheat growing areas are affected by waterlogging each year [1], especially in the irrigated rice-wheat growing environments of south and southeast Asia [2].

Waterlogging negatively affects the root system, which restrains the growth of plants, and eventually affects the yield of crops [3, 4]. Hypoxia, nutrient deficiency, and microelement toxicities are considered as the main factors caused by waterlogging. Severe hypoxia or anoxia in the root zone is the most serious factor [5, 6]. When plants are transferred from aerobic respiration to anaerobic respiration under low oxygen conditions, low availability of ATP slows down the growth and metabolism [7]. Even though stress responses may occur in the early stages of hypoxia, such as the formation of aerenchyma, root cells will remain in a hypoxic state. The death of these cells often leads to the abscission of some roots [8]. The decrease in water and nutrients absorption results in lack of nutrition and dehydration in tissues above the ground [9]. Stomatal closure of leaves occurs as a result of dehydration and causes reduction in intercellular carbon dioxide concentration. Inhibition of photosynthesis leads to a decrease in the accumulation of dry matter production in crops [10]. In addition, the denitrification of organic and inorganic soil nitrogen caused by waterlogging, reduced the leaf photosynthesis [11].

Significant differences in the tolerance to hypoxia stress exist among wheat genotypes [12]. Under hypoxia, tolerant genotypes were found to be better in root growth [13] and morphological adaptations [14], such as the formation of more aerenchyma compared to sensitive genotypes [13]. Furthermore, tolerant genotypes maintained higher physiological and metabolic activities than sensitive ones under waterlogging stress [14–16]. A lot of QTLs associated with waterlogging tolerance in wheat have been identified [17–19].

Proteomic analyses have been successfully used to study different stresses responses, such as salt stress [18, 20, 21], drought stress [22, 23], heat stress [24–26] and waterlogging stress [27–31] in various crops. Energy reduction inhibits the synthesis of protein induced by anoxia, leading to changes in gene expressions and protein expression patterns. *TaBWPR-1.2#2*, an important gene related to waterlogging tolerance, was reported to regulate the function of metabolic adjustment [32]. Proteins associated with energy metabolism and stress defense were shown to be waterlogging responsive in wheat [15]. Protein abundances related to primary metabolism and signal transduction changed in the early stages of hypoxia, such as UDP - glucose dehydrogenase, UGP, beta glucosidase [33]. Furthermore, Defense proteins [34], such as glycosylated polypeptide, alpha amylase, and signaling proteins [35], such as phosphatidyl ethanolamine binding proteins (PEBPs), were up-regulated after waterlogging treatment. However, most of the previous studies used only one genotype to analyze proteomic changes due to waterlogging stress. In this study, two wheat genotypes contrasting in waterlogging tolerance were selected. The Tandem Mass Tag (TMT) labeling-based quantitative proteomic analysis was conducted parallel on these two genotypes in response to hypoxia for different time points. Further studies were also conducted to confirm the involvement of several selected genes in waterlogging tolerance. This will potentially enable us to reveal the regulatory mechanisms of waterlogging tolerance in wheat.

## Results

### Impact of waterlogging on wheat seedling growth

**Fig. 1:**
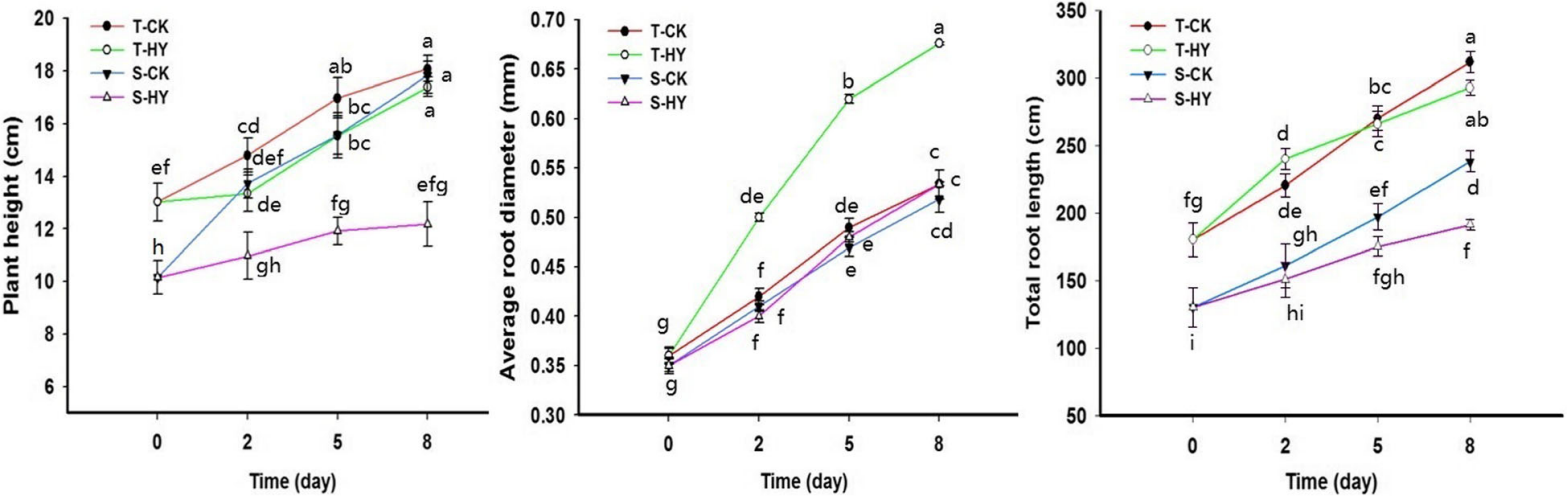
Effects of hypoxia treatment on the growth of two wheat varieties. X-axis stands for the time of treatment; T and S stand for the tolerant and sensitive varieties, respectively. Different letters indicate significant level (*P* < 0.05). Means ± SE (*n* = 30)

To show the difference in waterlogging response, hypoxia treatment was performed to mimic the waterlogging condition during wheat seedling growth in both S and T. As expected, hypoxia stress negatively affected the growth of both cultivars (Fig. 1). However, the S and T responded differently to the hypoxia treatment (Fig. 1). The plant height of S was reduced by 20.02, 23.33 and 31.69% at 2, 5 and 8 days of hypoxia stress, respectively, while those for T were only 9.80, 8.37, and 3.82%, respectively. It is obviously that hypoxia could reduce the growth of both cultivars within 5 d. The average diameter of roots increased significantly in T (26.6%), but there was no obvious difference in S after hypoxia treatment. The total root length significantly increased by 8.8% at 2 d, while it reduced by 6.1% at 8 d in T. But it reduced from 0 d to 8 d after hypoxic treatment in S (Fig. 1).

**Fig. 2:**
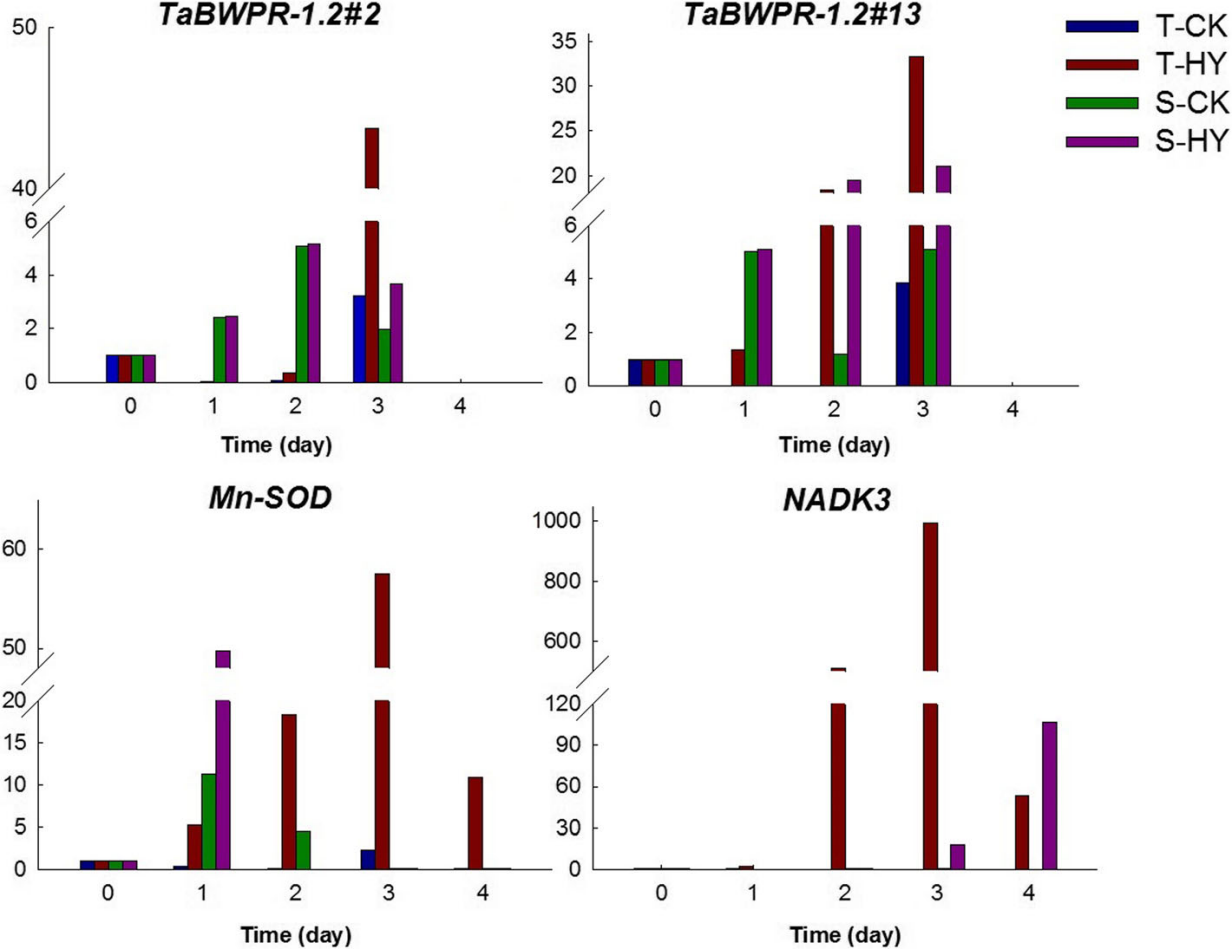
Expression of some water-logging responsive genes at mRNA level during the treatment. Y-axis stands for the relative mRNA level, X-axis stands for the time of treatment

To keenly track the response at molecular level, the expressions of representative hypoxia genes were analyzed at different time point of treatment. *TaBWPR-1.2#2* and *TaBWPR-1.2#13* are two genes verified to be related to hypoxia response and aerenchyma formation in wheat roots [32]. Here, we first detected the expressions of the two genes in treated wheat. *TaBWPR-1.2#2* showed a significant increase only in T after 3 d of hypoxia treatment (Fig. 2). In contrast, *TaBWPR-1.2#13* was up-regulated in both genotypes after 2 d of hypoxia treatment (Fig. 2). Meanwhile, the expressions of other two stress responsive genes *Mn-SOD* and *NADK3* were also analyzed during the treatment. The expression level of *Mn-SOD* gradually increased in the tolerant genotype with prolonged duration of hypoxia treatment until 3 d after hypoxia treatment. However, its expression in the sensitive genotype S increased sharply after 1 d of treatment, but decreased quickly and could be hardly detected with prolonged treatment (Fig. 2). As for *NADK3*, it was up-regulated by hypoxia stress only in T (Fig. 2). Based on the changes of seedling growth and genes expression, the plants at 0, 1, 2 and 3 d after treatment were used for further proteomic analysis in this study.

### Changes of protein profiles in two cultivars during hypoxia treatment

**Fig. 3:**
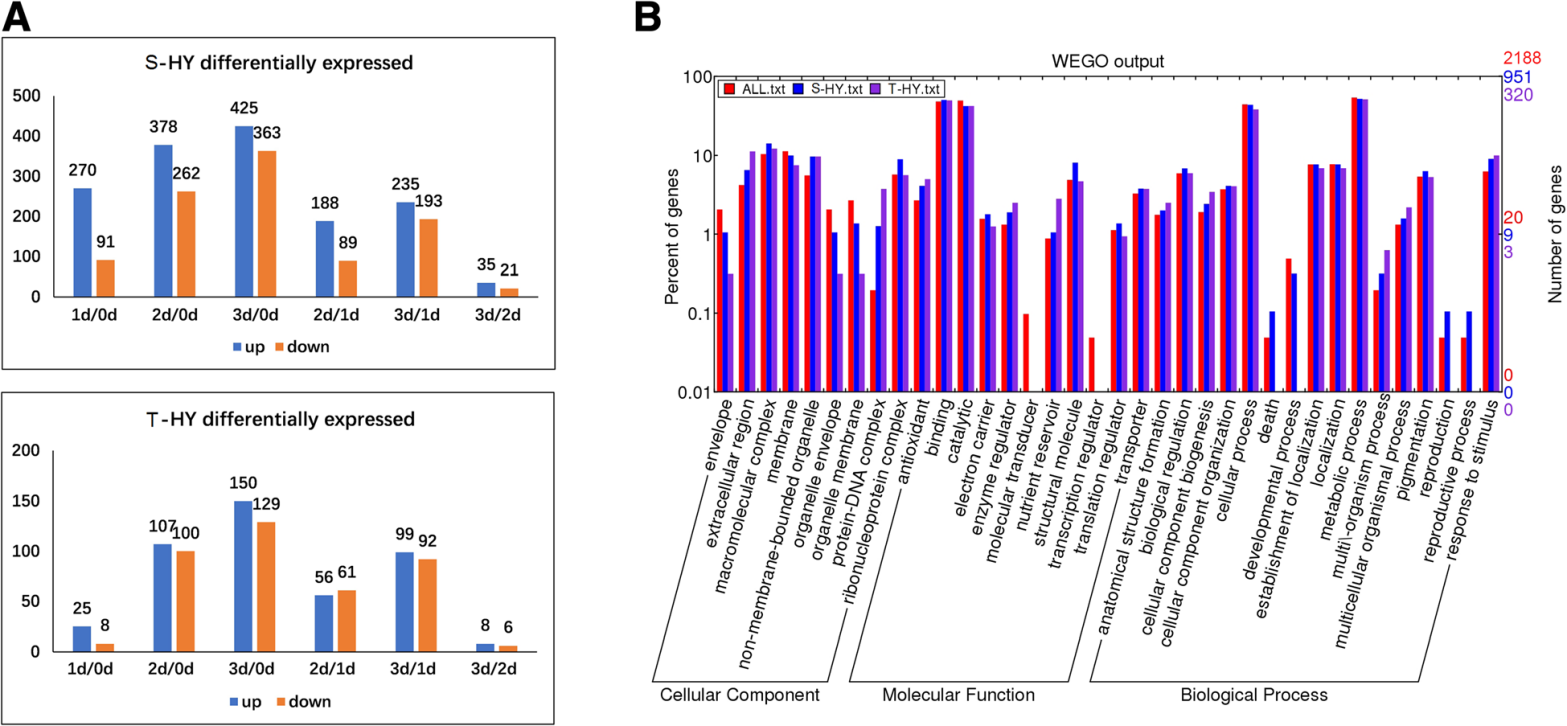
The statistic and GO annotation of the DEPs in the two wheat varieties. (**a**) Numbers of differentially expressed proteins (DEPs) in S and T varieties. Y-axis stands for the number of DEPs, X-axis stands for the comparisons between different time of treatment. (**b**) GO annotation of the whole identified proteins and DEPs in S and T varieties. All stands for the total identified proteins, S and T stand for the DEPs identified in S and T varieties, respectively

To explore the underlying mechanisms that lead to different hypoxia responses in the two wheat genotypes, quantitative proteomic analysis was conducted on roots of both varieties. After quality control filtering (Additional file 1: Figure S1), a total of 4007 and 4010 proteins were quantified in all samples of S and T, respectively with a high degree of reproducibility (Additional file 2: Figure S2). Among these quantified proteins, 2188 that appeared in all three replicates were used to search the differentially expressed proteins (DEPs) during the treatment. Much more DEPs were detected in S (951) than that in T (320) with folds change > 1.5 (*p* < 0.01, Fig. 3a, Additional file 3: Table S1). The number of DEPs was 361 in S in the first day of treatment, but was only 33 in T (Fig. 3a).

Gene Ontology (GO) annotation showed that these DEPs covered all cellular components, biological processes and most molecular functions (Fig. 3b). When comparing between the two cultivars, no DEPs involved in death, development process, reproduction and reproductive process were detected in the T genotype in terms of biological process (Fig. 3b).

**Fig. 4:**
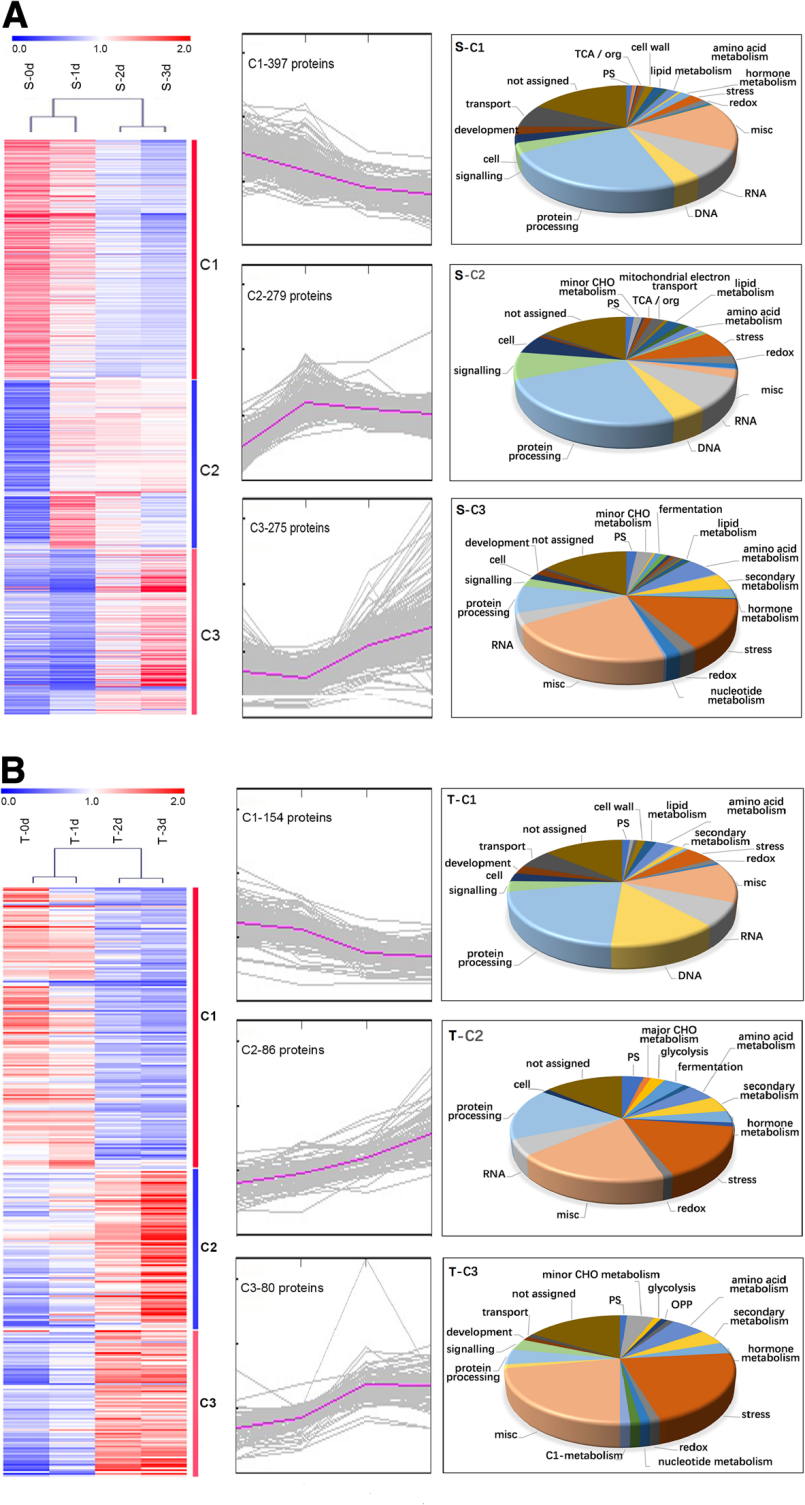
Clustering and the function classification of the DEPs. (**a**) S variety; (**b**) T variety. Left, central and right panels show the hotmap, K-means clustering and Mapman functional classification, respectively

To further understand the possible roles of the DEPs in wheat waterlogging response, we clustered the DEPs on the basis of the abundance changes at different time points using K-mean method in S and T, respectively. In both cultivars, all DEPs were divided into 3 clusters (C1-C3, Fig. 4, Additional file 4: Table S2). For S, C1 contained 397 (42%) proteins, and showed a gradually decreasing pattern during treatment. Protein processing, misc., RNA processing and transport related proteins were the main functional groups in this cluster (Fig. 4a). C2 contained 279 proteins and the abundance of 24% proteins peaked at the first day of treatment. Protein processing, RNA processing, stress, and signaling related proteins were the main groups (Fig. 4a). C3 contained 275 proteins, and 65.5% proteins in this cluster did not change at 1 d of treatment and increased along with the treatment after 2 d. Misc, stress and protein processing related proteins were the main groups (Fig. 4a). For T, C1 contained 154 proteins (48%), and showed a decreasing pattern with a sharp decrease at two-day-treatment. Protein processing, misc., DNA and RNA processing related proteins were the main groups in this cluster (Fig. 4b). C2 contained 86 proteins, and all proteins gradually increased along with the treatment. Protein processing, misc., and stress related proteins were the main groups (Fig. 4b). C3 contained 80 proteins, and showed an opposite changing pattern with C1. Misc and stress related proteins were the two main groups (Fig. 4b).

**Fig. 5:**
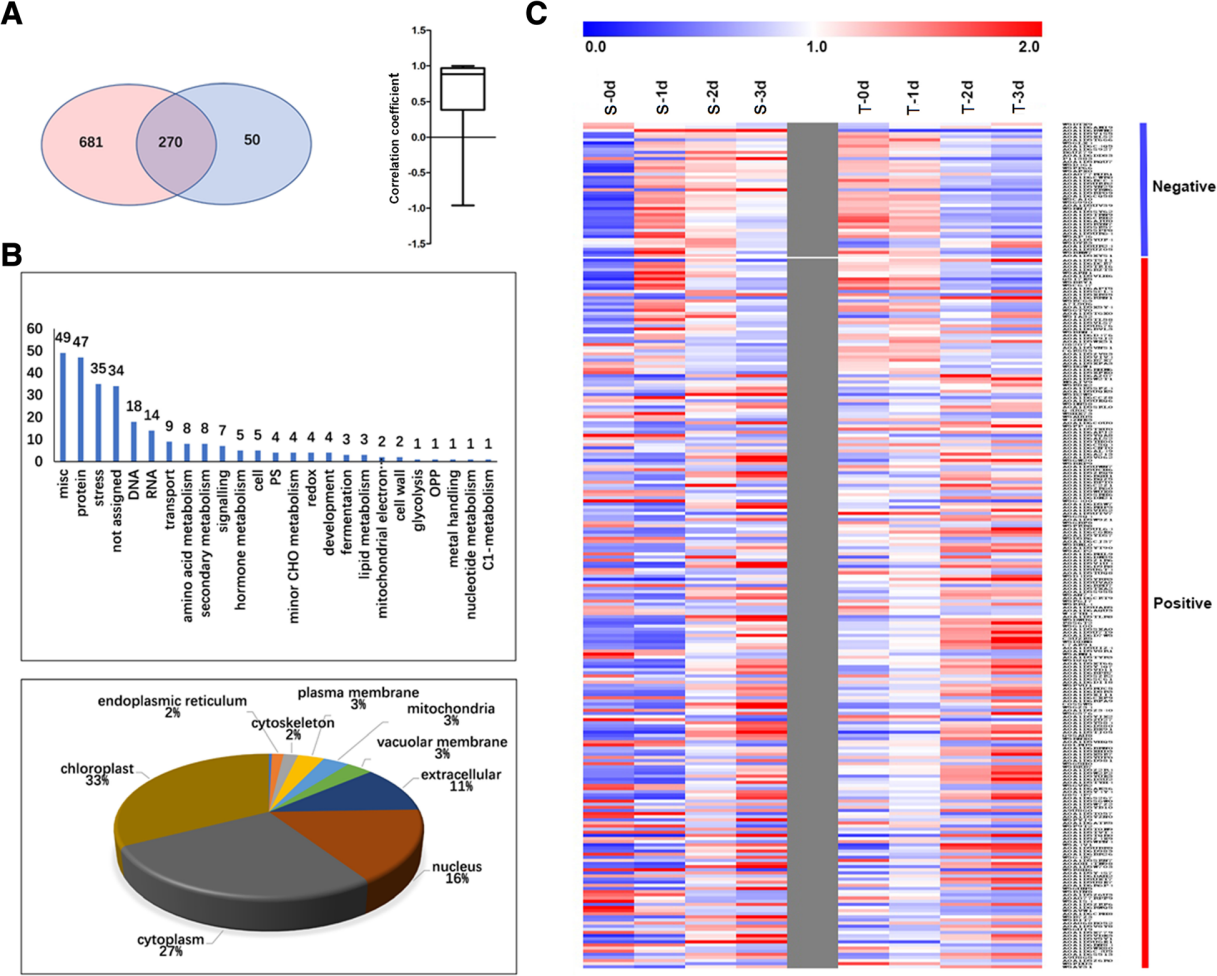
Comparison of common DEPs in S and T varieties. (**a**) Venn diagram of the DEPs between S (left) and T (right) varieties, and the correlation coefficient between the accumulation of the shared DEPs from the two varieties (right panel). (**b**) Function classification (Up) and subcellular location of the shared DEPs (Down). (**c**) Hot map showing the expressional patterns of the shared DEPs in S and T

Comparison between the DEPs of S and T showed that 270 proteins commonly existed in both cultivars, of them, 77% having similar expressional patterns during treatment (Fig. 5a). Most of these common proteins were misc. (18.1%), protein processing (17.4%), stress (13.0%), and DNA (6.7%) and RNA (5.2%) processing related proteins (Fig. 5b). Among them, 33, 27, and 16%were located in the chloroplast, cytoplasm, and nucleus, respectively. (Fig. 5b). However, there were still 43 proteins showing opposite expressional patterns between the two cultivars, most of which increased in S but decreased in T in response to hypoxic stress, especially for DNA and RNA processing and signaling related proteins (Fig. 5c).

**Fig. 6:**
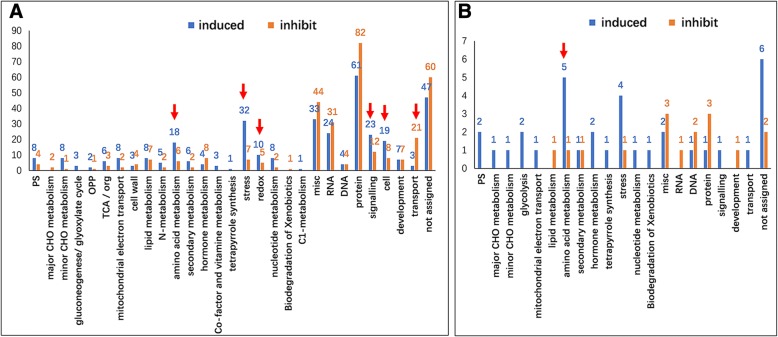
Functional categorization of the S and T specific DEPs. (**a**) S-specific DEPs; (**b**) T-specific DEPs. Y-axis indicate the number of proteins. Arrows indicate the enriched groups

Since the two cultivars showed different hypoxia tolerance, the cultivar specific DEPs in S and T were also analyzed. There were 681 S-specific and 50 T-specific DEPs (Fig. 5a). Among the 681 S-specific DEPs, 45.5% of them are protein processing, misc., stress, RNA processing and signaling related proteins, and 355 were hypoxia induced (Fig. 6a). The number of proteins involved in stress and redox, signaling, cell organization and amino acid metabolism were greater in the induced group than that in the decreased group (Fig. 6a). Notably, 20 heat-shock proteins (HSPs), in particular HSP70, were significantly induced. For signaling related proteins, the 14–3-3 and calcium binding proteins were remarkably increased, but G-proteins, e.g. ras-related proteins, were reduced. It is interesting that 12 ubiquitin proteome system (UPS) related proteins were increased as well. Whereas, the transport related proteins were enriched in the decreased group, especially the aquaporin, such as tonoplast intrinsic proteins. Among the 50 T-specific DEPs, the amino acid metabolism and stress related proteins were also induced by hypoxia (Fig. 6b).

### RNA expression levels of waterlogging specific response genes in wheat seedling

**Fig. 7:**
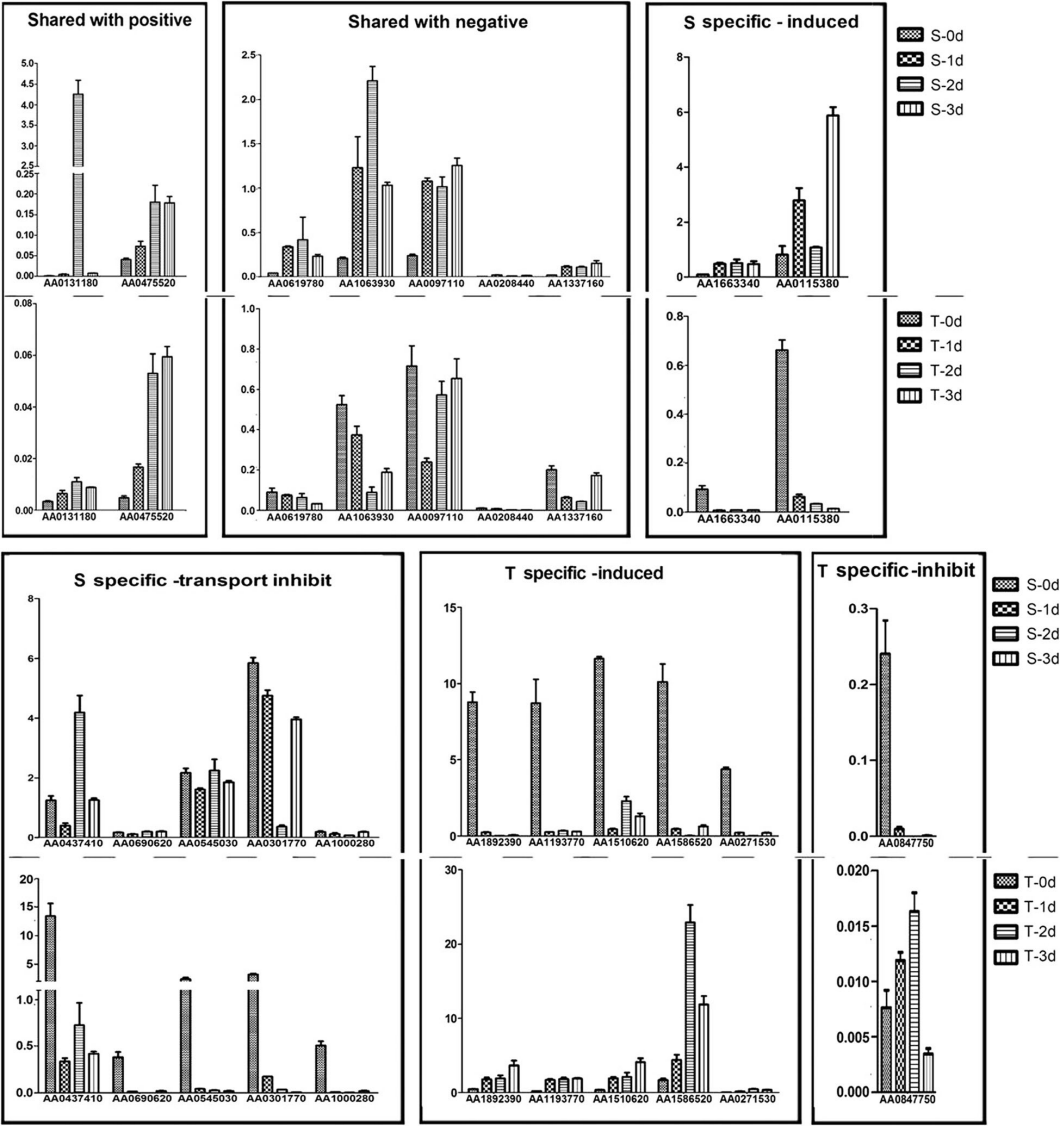
Verification of the DEPs encoding genes’ expression at mRNA level. Y-axis stands for the relative mRNA level, X-axis stands for different DEPs

To further explore the regulation of the genes encoding the DEPs, a total of 20 representative genes were selected for RT-qPCR analysis (Fig. 7), including 7 shared DEPs (2 with similar patterns and 5 with opposite patterns), and 13 cultivar-specific DEPs (7 increased and 6 decreased). The expression of 14 genes showed consistent dynamic patterns at mRNA and protein levels. However, some decreased proteins encoding genes were up-regulated at mRNA level. For example, five aquaporin tonoplasts intrinsic protein (TIPs) decreased significantly in S at protein level, but were up-regulated at their mRNA level with the prolonged waterlogging time. Interestingly, the mRNA levels of all these TIPs decreased rapidly in T during treatment.

## Discussion

### Wheat responses to hypoxia treatment at morphological and physiological levels

Oxygen shortage due to waterlogging is one of the main factors causing a negative effect on wheat growth, especially at the seedling stage [8]. Differences in waterlogging tolerance exist in wheat genotypes. The two cultivars used in this study responded to hypoxia stress very differently at both growth and molecular levels (Figs. 1 and 2), confirming the higher tolerance of CIGM90.863-SH64 to waterlogging [36]. Under hypoxia stress, fewer changes in protein expression patterns were found in the tolerant cultivar compared to the sensitive one (Fig. 3, Additional file 2: Figure S2).

### General adaptations of wheat seedling in response to waterlogging

Upon the waterlogging treatment, significant changes at morphological and proteome levels started after the first day of the treatment in S while the changes were shown after two days of the treatment in T. The anaerobic adaptations were the common responses in the root cells of both cultivars, which is supported by the results from this experiment that many anaerobic adaptation related proteins such as 4 alcohol dehydrogenases (ADH) and 3 fermentation related enzymes were significantly increased during stress. Deficiency of oxygen caused an inhibition of primary metabolisms, including the amino acid synthesis and lipid metabolism [37]. In this study, a majority of the protein degradation (proteasome and protease related) related proteins increased, whereas, many protein processing (protein synthesis and targeting) related proteins decreased dramatically after waterlogging treatment. DNA and RNA processing (Histones and transcription regulators), and transport related proteins were also significantly reduced after hypoxic stress, possibly due to the lack of energy in root cells to work on the synthesis of nucleic acid and proteins by anaerobic respiration, similar to that reported in maize root [19]. This indicates that waterlogging could inhibit some transcriptional and translation processes and seriously affect the stability of proteins. Interestingly, the secondary metabolism related proteins, e.g. 5 isoprenoids related proteins that participate in the terpenoids synthesis, were significantly increased. Further studies could be conducted to explore the mechanisms for the enhancement of terpenoids synthesis functions in wheat responding to waterlogging.

Under hypoxia stress, both biotic and abiotic stress related proteins were rapidly increased. These proteins include pathogenesis-related proteins, peroxidases and glutathione S transferases, and HSPs. Similar results have also been detected in many other plants, such as soybean, maize, cucumber [27–31, 38]. Altogether, waterlogging could inhibit primary metabolisms and substance transportation, negatively affects protein stability, and initiates the general stress responsive processes.

### Mechanism underlying waterlogging tolerance in wheat

Except for the common adaptions to the hypoxia stress, cultivar-specific responses also could provide some insights into the pathways involved in the sensitivity or tolerance of wheat under waterlogging. Three major aspects revealed by our data may have contributed to wheat waterlogging tolerance enabling long-term survival: the production and consumption of energy; the production of glutathione (GSH); and the metabolism process.

A reduced production and increased consumption of energy are main restrictions for plant growth [39]. Under hypoxia condition, glycolysis pathway produced harmful substances such as lactic acid and acetaldehyde, which could be reduced by alcohol dehydrogenases and fermentation related enzymes [27, 29, 30]. Moreover, low degree of glucose metabolism, ATP and NAD+ formation in ethanol fermentation play important roles in helping root cell survival from hypoxia stress. In our study, the abundance of three alcohol dehydrogenase (A9U8G0, W5A4V1, A9U8G5) were increased more quickly in T than that in S, and another alcohol dehydrogenase (A0A1D6DDI9) was reduced only in S by 47.2%. For some metabolic reactions of energy consumption, several proteins related to amino acid, DNA and RNA processing (W5GLX4, A0A1D6DD83, W5CAI0, A0A1D5SY62) maintained a high abundance at the beginning of the treatment, but showed continuous decrease in T and increase in S from 0 d to 3 d after the treatment, indicating more energy consumption in S than T [40]. There were 355 up-regulation and 326 down-proteins in S while only 34 and 16 in T, suggesting that T had a better ability to maintain protein stability and more efficient energy acquisition and utilization after hypoxia stress.

Glutathione (GSH) plays an important role in maintaining the function of immune system by its ability in cleaning reactive oxygen species (ROS) in plant cells under hypoxia stress [41]. It was reported that glutathione was regarded as an important protective enzyme in several crops under waterlogging stress. In this study, 6 shared proteins related to glutathione transferase were increased greatly in T compared to S. The expression levels Mn-SOD and NADK3, which are responsible for ROS cleaning [41], increased significantly in T. In this case, T showed a strong ability to avoid oxidation of cytomembrane, which could be one of the key contributors for its waterlogging tolerance.

The metabolism process, including the lipid metabolism, amino acid metabolism, secondary metabolism, was more active in T than that in S, except for the photosynthesis (PS). The enhancement of primary metabolism is a short-term strategy for plant to escape from waterlogging stress [42]. T Showed a better tolerance through maintaining the expression of stress related genes, including kiwifruit pyruvate decarboxylase 1 gene (*AdPDC1*) which is reported to enhance waterlogging tolerance in *Arabidopsis* [43]. The reduced expression of HvPRT6, an E3 ligase in T contributed to its enhanced waterlogging tolerance [44]. Two proteins (C1K737 and W5EPH8) related to ethylene metabolism, a key mechanism for aerenchyma formation, decreased only in S. Similarly, higher expression level *Ta-BWPR1.2#2* and *Ta-BWPR1.2#13* in T lead to a better ability in obtaining oxygen in the root cell [13]. More than 30 proteins showed higher expression levels in T than that in S during the whole process of treatment. These proteins are mainly biotic stress related proteins, including the pathogenesis-related family proteins, jasmonate-induced proteins. In addition, most of the 34 T-specific DEPs were metabolism and stress related proteins. Among them, S-adenosylmethionine synthetase 1 (SAM1, AA1586520) is a protein that creates S-adenosylmethionine by reacting methionine and ATP, participates in the DNA methylation, ethylene biosynthesis and other methylation reactions [45]. SAM1 is preferentially expressed in all vascular tissues, stem sclerenchyma and root cortex, and can be induced by several stresses, e.g. NaC1, mannitol and ABA treatments [46]. In this experiment, SAM1 was also induced in T at the second day of treatment, indicating that the SAM1 might also be involved in the hypoxia tolerance in wheat.

In this study, 14 genes showed consistency in transcription and translation level, but 6 genes were inconsistent with the transcription and translation level. In fact, this phenomenon had ever been detected in many researches [47, 48]. There are many factors affecting the regulation of gene expression. Besides regulation of transcription level, post-transcriptional regulation, translation and post-translational regulation all play important roles in the final protein expression. Furthermore, the degradation of RNA, mRNA selective transcription, and protein degradation, modification and folding may lead to the inconsistency between the abundance of RNA and the expression level of protein, for example, miRNA can inhibit protein expression or directly degrade mRNA. This imbalance also emphasized the post-transcription level regulation in seedling stress.

### Potential protein markers for waterlogging stress

In recent years, molecular marker assisted selection has been widely used in crop breeding, which shortens the time of breeding by direct selection of the target traits [49]. As biomarkers, proteins are more diverse, and show more direct and dynamic response to internal control of plants, which has great application prospects in cultivar screening [31]. In this study, increased expression of 34 proteins, including alcohol dehydrogenases and SAM1, was evidenced in the tolerant genotype under hypoxia stress. Moreover, 51.9% of T-specific proteins significantly increased in the 3 d after the treatment. These proteins may be used as candidate biomarkers for waterlogging tolerance screening. Further, the effective biomarkers could be extensively used to in other cultivars screening.

## Conclusions

Waterlogging is one of the main global abiotic stresses limiting crop production, especially for the drought crop wheat. In this study, using two wheat genotypes (Seri M82, sensitive to waterlogging stress and CIGM90.863, tolerant to waterlogging) contrasting in waterlogging tolerance, we first mimicked the waterlogging condition and analyzed the impact of waterlogging on different wheat seedling growth. Then, using TMT labelling technique, we investigated the dynamic response of two wheat genotypes to hypoxia stress at proteomic level. Primary metabolisms and protein processing were found easily be affected and degraded under hypoxia stress, especially in the sensitive genotype. Some important proteins, such as acid phosphatase, oxidant protective enzyme, SAM1, maintained higher expression levels in the tolerant genotype than the sensitive one. These proteins might be used as biomarkers for waterlogging tolerance screening in wheat or other crops.

## Methods

### Wheat genotypes, waterlogging treatment and phenotypic data collection

Seeds of two spring-type wheat genotypes (Seri M82 and CIGM90.863) were collected from International Maize and Wheat Improvement Center (CIMMYT). Seri M82 (hereafter, named as S) is a relatively waterlogging sensitive genotype, while CIGM90.863-SH64 (hereafter, named as T) is relatively tolerant to waterlogging [36].

The seeds of both genotypes were germinated and cultivated in a seedling tray for 10 days. Healthy plants with a similar height were transferred to plastic containers (100 × 150 × 60 cm, height × length × width) containing 200 L Hoagland nutrient solution with oxygen delivered by air pump. Each container held 30 plants of the two genotypes. Two days after transferring when the plants adapted to hydroponics, half of the containers were subjected to hypoxic treatment by bubbling N_2_ gas using automatic oxygen meter (America, Quantum) and the oxygen content of water was controlled at 2.0 mg/L. The other half were used as controls and provided with sufficient oxygen with an air pump. The experiments were carried out in a greenhouse at 22 °C/18 °C (day/night). A completely randomized design (CRD) with three replicates was applied in this experiment.

### Gene expression profile analysis

Forty plants of both genotypes were transplanted into 20 growth boxes (20 × 10 × 10 cm, height × length × width) with 2 L of Hoagland nutrient solution after growing in seedling tray for 10 days. These growth boxes were then put into a growth chamber under controlled conditions (16 h light/8 h dark cycle at 22 °C/18 °C day/night temperature, relative humidity 60%). Oxygen was provided by an air pump. Two days later, 10 growth boxes were adjusted to a stable hypoxia environment with oxygen content at 2.0 mg/L, which was achieved by adding 0.1% agar [50] and bubbling with N_2_ gas for 30 min.

Tissue samples for RT-qPCR were collected in 1st, 2nd, 3rd and 4th days after treatment. The plants were taken out of nutrient solution, the roots were excised from each plant and sealed with silver paper and then flash-frozen in liquid nitrogen immediately.

Total RNA was extracted with extracting agent (TRIzol) and detected using denaturing agarose gel electrophoresis. The quality and concentration of total RNA were determined using the NanoDrop (NanoDrop 2000C). Reverse transcription was performed with Reagent kit (Takara) as per the instructions. Assays were carried out with the QuantStudio 6 (ABI) using SYBR™ green PCR master mix (ABI), and the primers in the above tests were as follows: the primers 5′-cttgacgccgaagcctagta-3′ (forward) and 5′-gccggaatgtgtgcttattt-3′ (reverse) for *TaBWPR-1.2#2*; 5′-cgcactggtcatagtcatgg-3′ (forward) and 5′-ctgttgtcccacgtcacag-3′ (reverse) for *TaBWPR-1.2#13*; 5′-tcccgctgttggatctttgtat-3′ (forward) and 5′-gtttattagcaacgcaggcaca-3′ (reverse) for *Mn-SOD*; 5′-tgcctgtgttttttatccga-3′ (forward) and 5′-accgtccatgtgcctgtagt-3′ (reverse) for *NADK3*; 5′-cagcaatgtatgtcgcaatc-3′ (forward) and 5′-tagcatgaggaagcgtgtat-3′ (reverse) for *β-ACTIN*.

### Protein extraction, trypsin digestion and TMT labeling

Proteins were extracted according to TCA precipitation method as described previously [51] from the roots of both cultivars after 0, 1, 2 and 3 days waterlogging treatment. Three biological replicates were applied and 24 raw data files with the fine mass accuracy were obtained (Additional file 5: Table S3 and Additional file 1: Figure S1). Briefly, the samples of root were ground in liquid nitrogen into fine powder. The powder was then transferred into a 5-mL centrifuge tube and mixed with four volumes (*v*/*w*) of lysis buffer (8 M urea, 1% Triton-100, 10 mM dithiothreitol, and 1% Protease Inhibitor Cocktail). After sonication for three times on ice with an ultrasonic processor (Scientz), the debris was removed by centrifugation at 20,000 g at 4 °C for 10 min. Finally, the proteins were precipitated with cold 20% TCA at − 20 °C for 2 h. After centrifugation at 12,000 g, 4 °C for 10 min, the pellet was washed with cold acetone for three times. The proteins were dissolved in lysis buffer (8 M urea, 100 mM TEAB, pH 8.0) and the protein concentration was detected with BCA kit (Bio-Rad protein assay, USA) according to the manufacturer’s instructions.

The protein solution was reduced with 5 mM dithiothreitol for 30 min at 56 °C and alkylated with 11 mM iodoacetamide for 15 min at room temperature in darkness. The sample was then diluted to urea concentration less than 2 M using 100 mM TEAB. Two-step trypsin digestion were performed, 1: 50 trypsin-to-protein mass ratios for the first digestion overnight and then 1:100 for a second 4 h-digestion.

The trypsin-digested peptides were desalted by Strata X C18 SPE column (Phenomenex) and vacuum-dried, and then were reconstituted in 0.5 M TEAB according to the manufacturer’s protocol for TMT kit. Briefly, one unit of TMT reagent (labeling 100 mg of protein) was reconstituted in 24 mL acetonitrile. The peptide mixtures were then incubated for 2 h at room temperature and pooled, desalted and dried by vacuum centrifugation.

### LC-MS/MS analysis and database search

Before mass spectrometry analysis, the peptide sample was fractionated into 18 using high pH reverse-phase HPLC with a gradient of 2 to 60% acetonitrile (CAN, Fisher Chemical) in 10 mM ammonium bicarbonate (pH 10.0) through Agilent 300 Extend C18 column (5 μm particles, 4.6 mm ID, 250 mm length). After vacuum dried and dissolved in 0.1% formic acid (FA, Fluka, solvent A), peptides were directly loaded onto a reversed-phase analytical column (15-cm length, 75 μm i.d.) with the gradient of solvent B from 6 to 80% at a constant flow rate of 400 nL/min on an EASY-nLC 1000 UPLC system. The detailed gradient of solvent B is 6 to 23% (0.1% formic acid in 98% acetonitrile) over 26 min, 23 to 35% in 8 min and climbing to 80% in 3 min then holding at 80% for the last 3 min. The peptides were subjected to NSI source, and the tandem mass spectrometry (MS/MS) were performed on Q Exactive™ Plus (Thermo) coupled online UPLC. The detailed LC- MS/MS parameters were referred to the previous report with minor modification [52]. Briefly, the electrospray voltage was 2.0 kV, the m/z scan range was 350 to 1800, and intact peptides were detected in the Orbitrap at a resolution of 70,000. For MS/MS, NCE was set as 28 and the fragments were detected at a resolution of 17,500, one MS scan followed by 20 MS/MS scans with 15 s dynamic exclusion. Automatic gain control (AGC) was set at 5E4.

The resulting MS/MS data were processed using MaxQuant search engine (v.1.5.2.8). Tandem mass spectra were searched against UniProt *Triticum aestivum* (136866) database concatenated with reverse decoy database. The default parameters were used with few modifications. Trypsin/P cleavage allows up to two missing cleavages. For precursor ions, the mass tolerance was set as 20 ppm in First search and 5 ppm in Main search, for fragment ions, that was 0.02 Da. FDR was adjusted to < 1% and peptides score was > 40.

The mass spectrometry proteomics data have been deposited to the ProteomeXchange Consortium via the PRIDE partner repository with the dataset identifier PXD008162. Username: reviewer51109@ebi.ac.uk. Password: POeI35ku.

### Bioinformatics methods

The proteins were classified into three categories: biological process, cellular compartment and molecular function by Gene Ontology annotation (http://www.ebi.ac.uk/GOA). MapMan (version 3.5.1, http://mapman.gabipd.org) was used to annotate protein function and pathway. The differentially expressed protein were evaluated by the two-tailed Fisher’s exact test with -Log(*P*) > 2. The heatmap was constructed using software MeV4.9 (http://en.bio-soft.net/chip/MeV.html). Correction for multiple hypothesis testing was performed with standard false discovery rate control methods *P* < 0.05.

### Statistical analysis

Treatment effects in the experiment were analyzed using one-way analysis of variance (ANOVA) procedure of SPSS software, version 14.0. Treatment means were separated by least significant difference (LSD) test at *p* ≤ 0.05 unless otherwise specified.

## Additional files

Additional file 1: **Figure S1.** Mass error and peptide length distributions of the MS data. (JPG 52 kb)

Additional file 2: **Figure S2.** Pearson correlation coefficient among three biological replicates of protein quantification. (JPG 196 kb)

Additional file 3: **Table S1.** The differentially expressed proteins (DEPs) (XLS 2273 kb)

Additional file 4: **Table S2.** Clustering of the DEPs using K-mean method (XLS 593 kb)

Additional file 5: **Table S3.** All identified proteins in this study (XLS 8212 kb)

## Abbreviations

ADH: Alcohol dehydrogenases
DEPs: Different expression proteins
HSPs: Heat Shock Proteins
ROS: Reactive oxy gen species
RT-qPCR: Time quantitative PCR
