# Supplementary figures and images for: Proteomic analysis reveals response of differential wheat (Triticum aestivum L.) genotypes to oxygen deficiency stress
Source: BMC Genomics. 2019 Jan 18;20:60. doi: 10.1186/s12864-018-5405-3 (PMC6339445; doi:10.1186/s12864-018-5405-3)

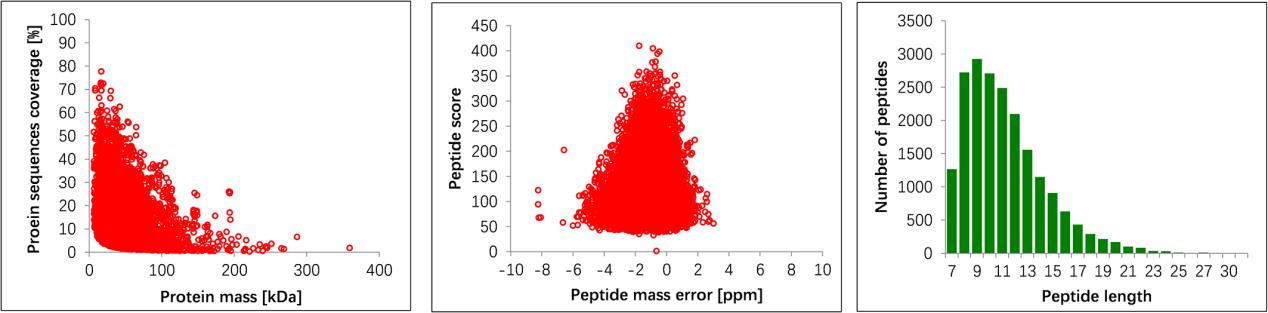

Supplement: Supplementary file 1 — Figure S1. Mass error and peptide length distributions of the MS data. (JPG 52 kb) [file 12864_2018_5405_MOESM1_ESM.jpg]

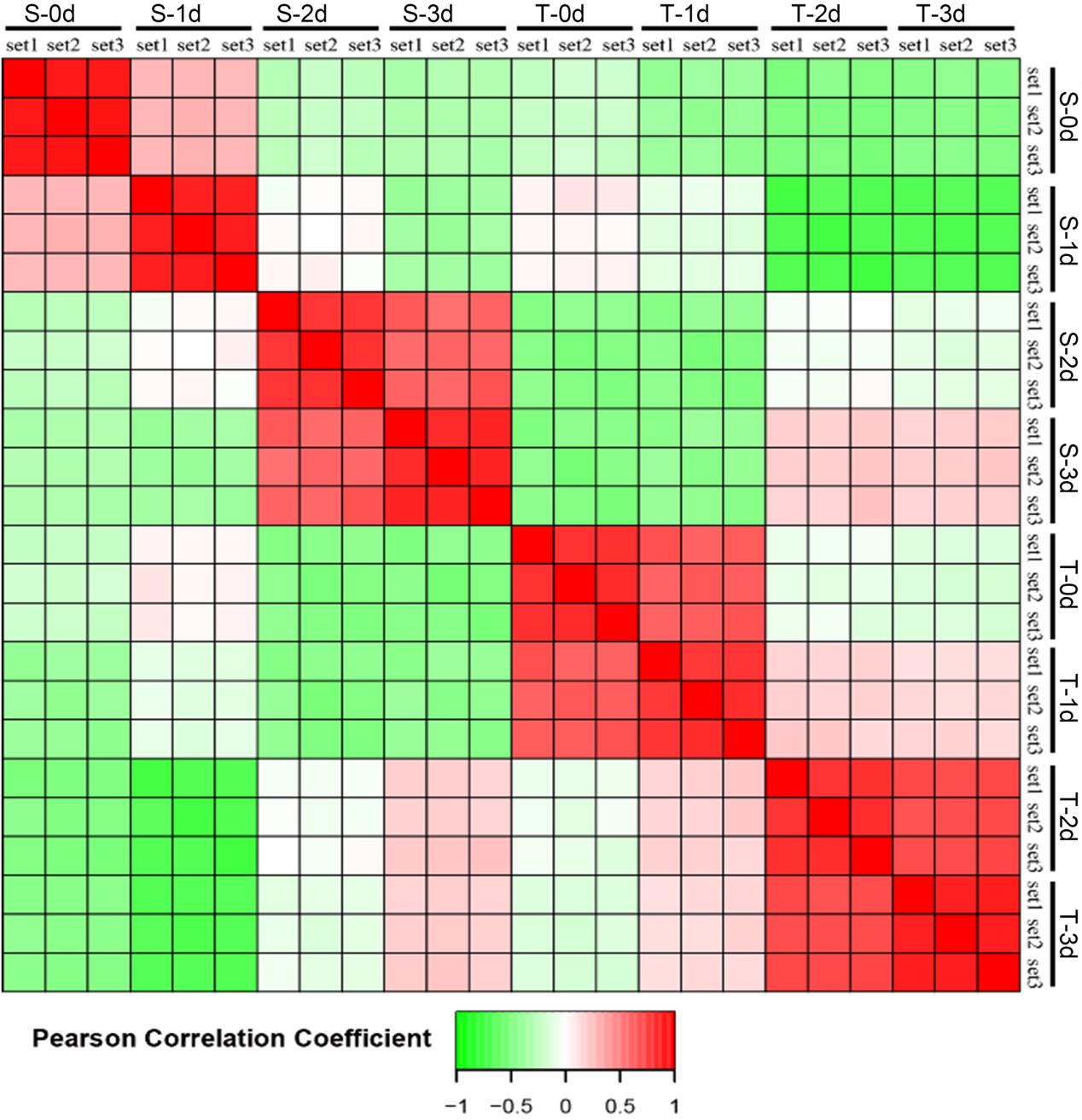

Supplement: Supplementary file 2 — Figure S2. Pearson correlation coefficient among three biological replicates of protein quantification. (JPG 196 kb) [file 12864_2018_5405_MOESM2_ESM.jpg]
